# Potentials of arts education initiatives for promoting emotional wellbeing of Chinese university students

**DOI:** 10.3389/fpsyg.2024.1349370

**Published:** 2024-03-05

**Authors:** Yanan Guo

**Affiliations:** Academy of Fine Arts, Xinxiang University, Henan, China

**Keywords:** arts education, wellbeing of students, emotional intelligence, positive psychological capital, self-efficacy, Chinese university

## Abstract

The present study explores the intricate dynamics influencing the self-efficacy of Chinese university students through the interplay of participation in artistic activities, positive psychological capital, and emotional intelligence. In the context of technological advancements and various challenges post pandemic, this study delves into the multifaceted aspects of university life, where arts education plays a pivotal role in addressing students' emotional needs. By integrating emotional intelligence with self-efficacy, this study underscores the positive impact of artistic engagement on self-efficacy, while emphasizing on the transformative power of these pursuits. Also, this study establishes that the optimism and resilience contribute to this relationship by considering the mediating role of positive psychological capital. The moderating influence of emotional intelligence in the complex dynamics between arts education and positive psychological capital is another concern, thereby emphasizing the nuanced role of emotional intelligence. With a structured set of questions that were administered to 673 participants with 93.61% recovery rate, this study performs the Cronbach's α-test, validation factor, and several related tests in *SPSSStatistics* 29.0, *bootstrap*, and *AMOS* 25.0 software. Current results shows the importance of a holistic approach in Chinese institutions. With a focus on promoting artistic engagement to enhance students' self-efficacy, this study determines the profound impact of arts education on students' overall wellbeing and educational experience. In conclusion, this research highlights the constructive impact of artistic engagement on the self-efficacy of Chinese university students. Chinese institutions should encourage a varied range of artistic engagements as a response to the contemporary challenges confronted by their students.

## 1 Introduction

The long-lasting influence of humanities, culture, and arts is prominent in improving the emotional intelligence (EI) and life quality of individuals. The multifaceted nature of human life demands various avenues to creatively express themselves through analytical thinking and cultivation of social-emotional competencies (Yeo and Suárez, 2022). Specifically, the role of arts education stands out as a cornerstone for promoting the overall growth and efficacy of students in today's world. Even though arts education is believed to be merely an extracurricular pursuit for a long time, researchers find the arts education to act as one fundamental building block of any well-rounded individual (Ran et al., 2022).

This outlook specially holds since human civilization is expected to experience profound transformation of technology with the rise of artificial intelligence (AI). Its influence is expected to reshape the educational paradigms across the globe in coming years. Questions arise about the impacts of AI on the EI levels of university students (Xu and Choi, 2023). Thus, this opens up a new door of research for investigating the potential consequences of the rise of AI on EI and the self-efficacy (SE) in Chinese university students. Besides, the COVID-19 pandemic adds another layer to these challenges, because this caused a considerable reduction of artistic activities by young adults (Wang et al., 2020).

Whereas university education is about imparting knowledge to effectively shape an individual, Chinese universities offer a multifaceted journey of physical, emotional, mental, and spiritual development to their students (Li et al., 2020). This is marked by a myriad of expectations extending beyond the academic realm. Chinese university students navigate the associated complexities by grappling with various facets, such as personal growth, societal pressure, and pursuit of career aspiration. Researchers find that students managing the stress effectively can easily adapt to changing circumstances and are more likely to achieve success in the long run (Kristensen et al., 2023).

While the resilience and adaptability arise as essential components to successfully navigate the university life (Vitale et al., 2023), arts education spanning across disciplines including music, dance, visual arts, and theater, is paramount to address aforementioned emotional and psychological needs. This emerges as a potent catalyst for nurturing creativity (Ermis and Imamoglu, 2019). Besides, those skills assist students in problem-solving, critical thinking, and collaboration. However, this study observes that traditional university education in China addresses solely the cognitive skills of learners. They sometimes fall short to cater to the emotional and spiritual dimensions of human existence.

On the other hand, young adults with high level of SE tend to approach academic challenges with more confidence and resilience (Martin, 2023). They belief in their own capabilities that additionally influence their motivation, learning strategies, and overall academic performances. In the university context, where students face diverse academic and social demands, SE turns out to be a key factor in determining how effectively they navigate challenges to achieve their educational goals. Moreover, SE extends beyond academics (Jin and Ye, 2022). This impacts various aspects of students' lives, including their choices, interpersonal relationships, and overall wellbeing. Understanding SE is essential for empowering educational experience of university students (Ran et al., 2022).

Besides, an efficient utilization of leisure time can yield a myriad of benefits, encompassing personal, physical, and psychological wellbeing (Wu et al., 2020). Concurrently, this enhances the interpersonal connections and significantly contributes to elevated SE and overall satisfaction of university students (Kausar and Ahmad, 2021). The nexus between physical and mental health emerges as pivotal, while influencing both engagement in diverse activities and overall life satisfaction. Robust physical health facilitates an increased participation in various pursuits, while augmenting the overall sense of contentment (Xu and Choi, 2023).

A number of existing articles emphasizes on the link between the positive psychological capital (PPC) and the participation in artistic activities (PAA) (Zhao et al., 2020). However, this study introduces a novel dimension by integrating EI with SE as the variable. This marks one crucial step in comprehending the complex relationship connecting PPC and EI with PAA. Furthermore, recent articles highlight the multifaceted challenges and opportunities for Chinese university education policies, with some scholars exploring alternative educational paradigms like the Waldorf school movement.

### 1.1 Research questionnaire

The present study intends to concentrate on the possibilities of arts education programs in aforementioned context, thereby centering around the following research questions:

- How does participation in various artistic activities influence the self-efficacy of Chinese university students? Also, extending beyond creativity and/or leisure, does PAA contribute to students' critical thinking, collaboration skills, and overall satisfaction?
- How does PPC mediate the relationship between PAA and self-efficacy? Also, to what degree does the attainment of PPC through various artistic activities contribute to the self-confidence of students?
- In what ways does EI impact the complex dynamics between PAA and PPC for Chinese university students, consequently impacting their overall engagement and wellbeing?
- How does various forms of arts education address the emotional and psychological needs of Chinese university students amidst challenges like technological transformations and COVID-19 pandemic?

## 2 Literature review

The current section reviews the existing literature covering some broad aspects as follows.

### 2.1 On students' participation in artistic activities

Psychological wellbeing of any person characterizes the happiness, general health and prosperity. This is about feel-good by doing properly in day-to-day life (Elliott and Gramling, 1990). Historically, Ryff (1995) developed the foundation of the psychological wellbeing theory. They delineated six interconnected yet distinct aspects of eudaimonic dimension. Those components were the positive relationships, self-acceptance, growth, environmental mastery, autonomy, and meaning of life, as described in a major investigation of Awan and Soroya (2021). A number of other recent articles highlights the positive impacts of PAA on the wellbeing of individuals, thus being a therapeutic aid for various demographics (Fancourt and Finn, 2019).

Recently, Zheng et al. (2019) found that there was a substantial body of research on tourism development related resident perceptions, while there was a notable gap in understanding residents' emotional responses. They sought to address this gap by employing cognitive appraisal theory. This aimed at uncovering the origins and outcomes of resident emotions in the context of tourism performing arts within the urban and rural communities both. Gibson and Ewing (2020) found that the fine arts would serve as a medium for cultivating students in emotions, thinking, morals, practical skills, and aesthetics in the realm of higher education. They noted that higher education institutions should prioritize fine arts education to enhance students' professional knowledge, artistic achievements. Their suggested method was aimed at contributing academic development along with character and morality building. Thus, their policy could be aligned with the broader goals of universities. Amidst the commencement of a fresh semester during COVID-19 out-break, Wang et al. (2020) investigated the changes in anxiety levels among Chinese students, who were yet to graduate. They took into account 1, 172 students from 34 provincial-level units. They could identify that students of Hubei could exhibit distinct anxiety patterns. Their results also emphasized a need for collective attention by society, universities, and families to address psychological wellbeing of those undergraduates during these challenging times. Zhao et al. (2020) aimed to explore any control of PPC on the entrepreneurial intention among Chinese university students. Particularly, they focused on mediating impacts of entrepreneurial capitals. They had showed that PPC could indirectly impact students' entrepreneurial intention through the classical social and financial capital. They emphasized the mediating role of conventional entrepreneurial capitals in elucidating the connection between PPC and entrepreneurial intention, with social capital exhibiting a stronger influence than financial and human capital.

Of late, Heutte et al. (2021) acknowledged Csikszentmihalyi's concept of flow to have a pivotal motivator for creativity, wherein individuals had found fulfillment due to engagement in enjoyable activities. They associated psychological wellbeing with various other health benefits, such as a robust immune system, improved sleep pattern, lower blood pressure, and an extended lifespan. Aesthetic experience that involved the perception of intrinsic pleasure through aesthetic objects, had played a crucial role in their investigation. Yeo and Suárez (2022) discussed the mental health on encompassing elevated levels of psychological, emotional, and social wellbeing, while they were coupled with the absence of any mental illness. The environmental variables of that article interacted with cognitive factors and thus facilitated the creative behavior. Jin and Ye (2022) discussed the component-wise model of creativity. They emphasized on the interconnected variables, such as domain skill, creativity-related process, and intrinsic plus extrinsic motivation. Very recently, Yao et al. (2023) defined on the involvement in extracurricular activities and investigated its connection to the health of college students. When creating academic courses, this study recommended that educators should take into account the possible effects of continuing extracurricular activities on the health of college students.

### 2.2 On students' positive psychological capital

PPC is a substantial predictor of positive results in several different aspects. In a notable study, Seligman (1998) pioneered the concept of positive psychology. He represented the positive psychology as a paradigm shift within psychology. He shifted the focus from solely addressing mental illness to emphasizing positive factors, individual adaptability, self-actualization, and personal growth.

In contrast to traditional psychology, Kern et al. (2020) found that positive psychology could place a greater emphasis on academic research and real-world interventions, thus enhancing the wellbeing of individuals. They suggested to employ the positive psychology in student guidance related activities. This would assist educators to effectively nurture students' strengths, talents, and positive emotions. Wang and Guan (2020) enhanced the understanding of English learning motivation. They put more emphasis on the psychologically demotivated college students. They suggested the teachers to address various interrelated factors to improve the motivation of those young learners. Thus, they differentiated between freshmen and sophomores. Whereas freshmen prioritized acquiring language knowledge, sophomores expressed a desire for active class participation and relevance in current textbooks. Those insights could offer practical implications for educators to tailor strategies based on students' evolving needs and challenges in English learning.

In a novel approach, Li et al. (2020) employed a mixed-method approach on basis of the positive psychology with the complex dynamic systems. They investigated interplay of foreign language enjoyment with associated classroom anxiety among 1, 307 students in China. Classroom anxiety was demonstrated to make a significant negative association with self-rated skill across all groups, whereas language enjoyment exhibited a positive correlation. They determined some similar relationships between real English achievement and those emotions other than the low achievement group. Their analyses indicated inverse correlations between them across different English achievement levels. Around this time, Wu et al. (2020) examined the correlation among characteristics of students, psychological resilience, and coping styles in the context of undergraduate students. They found that female medical students were interested to adopt more positive coping styles. They suggested that enhancing psychological resilience through education and health promotion programs would contribute to foster positive coping styles while improving mental health for those undergraduates. Fellow researchers described that the positive psychology with positive state would provide a valuable lens for understanding mental wellbeing. The incorporation of PPC into adolescent education would emerge as a proactive approach to foster the positive psychological states among students.

Lately, Koydemir et al. (2021) discussed a theoretical framework that was categorized into three dimensions, namely trait, positive state, and unit. Whereas positive state encompassed the satisfaction, positive emotion, happiness, and love, they identified the positive trait to encompass enduring behavioral elements, including courage, wisdom, and endurance. In an extension of Seligman's framework, Xu and Choi (2023) considered a pivotal concept within positive psychology, namely the positive psychological capital (PPC). PPC comprised four measurable factors that were intricately linked to positive cognition of an individual. This could make PPC to be referred as a widely influential idea. PPC signified a positive psychological state owing to the dynamic interplay of those four factors. Also, each of those factors could be cultivated and managed through personal efforts and diverse learning systems. Chen et al. (2023) conducted novel investigations on the relationship between first-year college students' past and present learning experiences as well as the correlation between their current level of learning engagement and the psychological capital and academic self-efficacy they had built up from previous learning experiences.

### 2.3 On students' self-efficacy

Self-efficacy centers on how well an individual believes to do a task in a particular circumstance. Classically, Martin (2023) discussed SE, which was referred to any individual's belief on the competence to successfully accomplish particular goal and/or influence a desired outcome. While the measurement of SE is broad, this is sometimes specific to a particular activity or within a specific domain.

Among recent studies, Kausar and Ahmad (2021) conducted an inquiry into the correlation between SE, psychological wellbeing, and stress over the performing arts students in Pakistan. They found that the female in performing arts exhibited higher stress levels compared to men. Their findings had practical implications for students, counselors, and therapists, thereby providing valuable insights related to the stress and psychological wellbeing of adult population in Pakistan. Ran et al. (2022) considered SE to be more than a demand control goal or psychological trait. Also, they described this as the ability to align goals with one's potential in any specific situation. Thus, this played a pivotal role in various psychological disorders. Like, behavioral aspects like sadness and dysfunctional anxiety would occur under the low SE. In a novel study, Schunk (2023) focused on the self-regulation of two types of motives: self-efficacy and attributions. They could describe the operation of self-efficacy and attributions. Kristensen et al. (2023) examined longitudinal relationships among academic SE, related stress, and psychological distress in Norwegian adolescents over 3 years of upper secondary school. Their investigation on school-based strategies could reveal that academic stress directly influenced psychological distress, whereas the academic SE could partly mediate those. Besides, the gender differences in their study showed that boys experienced stronger interpersonal effects, while girls exhibited stronger individual impacts. Also, Motamed-Jahromi et al. (2023) studied on the promotion of self-care behavior among older adults suffering from type 2 diabetes. They employed a three-arm cluster randomized controlled trial in that study.

### 2.4 On students' emotional wellbeing

University students often face various stressors throughout their academic journey that impact their emotional wellbeing and academic advancement both.

In this regard, Schoeps et al. (2019) investigated the effects of a 2-month emotional education program in a study with 250 university students. The program significantly improved the emotional intelligence and subjective wellbeing immediately after completion, although not sustaining at follow-up. This indicated hardly any potential benefit of implementing some intervention programs for students' emotional wellbeing and subjective wellbeing. Zheng et al. (2020) investigated the relationship between parent-child relationships and emotional wellbeing among Chinese junior high school students taking private tuition. Tutored students reported higher levels of self-confidence and improved parent-child relationships. However, beyond a certain threshold of tutoring intensity, the positive effects gradually declined. Around this time, Vitale et al. (2023) conducted an exploratory study on Italian nursing students' psychological scenarios during the pandemic COVID-19, whereas Kohls et al. (2021) conducted an online survey among university students in Germany. They assessed mental health, COVID-19 attitudes, and pandemic-related social and emotional aspects. They found that during the pandemic, depressive symptoms were significantly associated with self-rated income changes and varied among faculties. A brief report on 295 students by Clabaugh et al. (2021) revealed heightened uncertainty about academic futures, significant stress, and coping challenges amidst COVID-19 disruptions. In that study, the female students reported poorer emotional wellbeing than males, while students of color experienced higher stress and uncertainty about academic futures compared to White students.

Very recently, Sandilos et al. (2022) explored some associations between the classroom implementation of a universal Social and Emotional Learning (SEL) program, teachers' emotional wellbeing, and teacher–student interactions. In their study, they could observe some interaction effects. This findings revealed that control group teachers with lower wellbeing would exhibit lower quality classroom organization, which was not observed for teachers in the intervention condition. Around this time, Pan et al. (2023) explored the impact of Chinese EFL teachers' affective scaffolding on the academic engagement and psychological wellbeing of 1,968 learners. They observed a positive correlation between psychological wellbeing and academic engagement. This established the importance of fostering a harmonious teacher-student relationship for the psych-emotional development of EFL students. Pozas et al. (2023) focused on a small-scale exploratory study examining Mexican students' perceptions of social inclusion, emotional wellbeing, and academic self-concept. Their results had put some lights on the socio-emotional aspects of inclusive education, thus emphasizing the need for further research and discussion on the implications of these results.

### 2.5 Objectives of the research

On basis of above deliberations, this research aims to comprehensively explore the intricate dynamics surrounding the impact of PAA on the self-efficacy of Chinese university students. This study shall investigate the mediating role of PPC in connecting PAA with self-efficacy. Also, this study explores how EI moderates the relationship between PAA and PPC, while evaluating the effects of artificial intelligence and pandemic like disruptions on the EI levels of Chinese university students. This study thus assess the broader impact of self-efficacy on various aspects of students' lives, emphasizing its significance in empowering the unique educational experience within Chinese universities.

## 3 Framing of hypotheses

### 3.1 Impacts of participation in artistic activities on positive psychological capital

Participation in artistic activities enhances individuals' psychological capital with increased self-confidence and resilience (Wu et al., 2020). The use of art therapy promotes a pleasant emotional and mental state of mind. Also, this greatly benefits individuals' mental health by allowing them to express the inner ideas through a variety of psycho-therapeutic activities. Therefore, various artistic activities improve the cognitive, emotional, and behavioral patterns of young adults (Park and Cha, 2023). This conceptualization underscores the intricate interplay between psychological resources and academic success within the educational context.

Many articles describe that the PAA has a favorable impact on university students' psychological wellbeing (Lavric and Soponaru, 2023). A variety of external factors, including positive and negative attachments, happiness, prosocial behavior, and excellent physical health, influence PAA. The favorable influence of PAA on psychological capital adds to the general psychological wellbeing of persons, who are engaged in creative endeavors. Even though the impact of integrity on academic outcomes is hardly immediate, researchers describe PAA to be a construct encompassing positive psychological resources. Thus, individuals characterized by high levels of integrity are more inclined to demonstrate elevated PPC, which in turn contributes to their improved academic performance (Wu et al., 2020).

Moreover, the notion of aesthetic experience that is typically derived from the appreciation of beautiful items, shows that any engagement with artworks and nature-based things considerably improves the physical and psychological wellbeing (Koydemir et al., 2021). This is consistent with the notion that higher education in any branch of arts assist students to grow in a variety of ways, such as emotions, thinking, morality, and values. All these observations exhibit that the creative pursuits of university students have vital role in their mental health. Creative activities can influence PPC that is needed to improve mental health. On basis of these deliberations, this study proposes the following hypothesis:

**H1:** Participating in artistic activities contributes positively to the formation of positive psychological capital among university students.

### 3.2 Role of students' EI between the participation in artistic activities and positive psychological capital

There lies a close relationship between artistic pursuits and PPC through EI. While this relation offers reciprocal benefits, there occurs an increased self-esteem through improved understanding of local relationships and a heightened sense of social impact (Vazquez-Marin et al., 2023). Participating in artistic activities, including music, offers pleasurable components that have been linked to stress reduction. The immersive experience provided by these activities becomes a source of enjoyment, creativity, and increased self-esteem, thereby positively impacting the emotional states of individuals (Wu et al., 2020). This underscores the therapeutic and stress-relieving aspects inherent in artistic pursuits, emphasizing their role in fostering a positive emotional landscape for young adults.

Engaging in three-dimensional arts programs, such as creating art, has a pronounced impact on the emotional states of university students. These activities foster enjoyment and creativity (Ermis and Imamoglu, 2019). Also, they lead to increased self-esteem and contribute significantly to the holistic development of students. Moreover, recent research underscores social impacts of cultural activities, specifically on developing EI among students (Xu and Choi, 2023). For example, participation in activities like dance has been shown to enhance teenagers' body awareness and sensory expressions. This highlights the diverse and positive outcomes of artistic engagement on the emotional and social facets of university students.

Researchers underscore the inter-connectedness of EI and PPC by identifying a significant correlation between them (Xu and Choi, 2023). Whereas the emotional control emerges to be a crucial factor in this relationship, the improved motivation influences organizational outcomes and productivity. University students with higher EI exhibit better stress management, conflict resolution skills, relationship-building abilities, and overall improved social lives (Wong and Law, 2002). The engagement in artistic pursuits, coupled with the cultivation of EI and positive psychological resources, contributes synergistically to the overall wellbeing of individuals. This underscores the importance of acknowledging the interconnections of these factors for a comprehensive perspective on personal and social development among university students. Aforementioned intricate relationships motivate the present study to propose the following hypothesis:

**H2:** Emotional intelligence plays a moderating role for university students participating in artistic activities with their development of positive psychological capital.

### 3.3 Role of students' self-efficacy between the participation in artistic activities and positive psychological capital

SE can be described as an individual's personal belief on the capability to achieve particular goals. This is a crucial aspect that can significantly impact a person's confidence level under various situations (Ran et al., 2022). In this regard, arts education plays a pivotal role in nurturing SE with the proper recognition of strengths. Arts education instills a sense of confidence to achieve both personal and societal goals. Thus, this defines an individual's personality by mastering the intricacies of social ascension, application, and intellectual progress (Özlem and Romanescu, 2020).

The significance of SE is evident due to its close association with avoidant behavior (Jin and Ye, 2022). Individuals with a positive physical self-assessment tend to be more secure in interpersonal relationships and achieve greater professional success. Conversely, those with a negative self-perception often experience feelings of unease, insecurity, and worthlessness throughout their lives (Ermis and Imamoglu, 2019).

In the context of extracurricular activities, participation in performing arts activities enhances the psychological wellbeing of university students (Kausar and Ahmad, 2021). This plays a crucial role in reinforcing SE of those young adults. Through music, dance, and drama, students face challenges that contribute to the development of their belief in their capabilities. This, coupled with the collaborative nature of performing arts, fosters essential social skills and thus creates an environment conducive to continuous SE reinforcement. In this scenario, this study frames the following hypothesis for Chinese university students that describe connections between their arts education and psychological wellbeing:

**H3:** Between participation in artistic activities and self-efficacy, positive psychological capital acts as a mediator for university students.

### 3.4 Role of students' EI between the participation in artistic activities and self-efficacy

In recent years, EI has emerged as a pivotal predictor of significant outcomes across diverse domains for university students, thus encompassing achievements in work, academic success, and graduate employability. Researchers have increasingly focused on exploring the multifaceted dimensions of EI by recognizing its potential implications for shaping the educational landscape (Wong and Law, 2002). There is a compelling argument to integrate relevant EI skills into educational curricula and design theoretically grounded training interventions tailored for young adults.

The conceptualization of EI varies among researchers, with some framing this as a cognitive ability involving the perception, use, understanding, and management of emotions (Lavric and Soponaru, 2023). Other researchers categorize EI as personality traits related to emotional handling, distinguishing between ability EI and trait EI. A recent addition to the discourse is the consideration of emotional self-efficacy (ESE), which reflects individuals' confidence in their emotional abilities. High ESE equips individuals to approach emotional challenges with assurance and competence, thereby contributing to overall psychological wellbeing (Zheng et al., 2019).

At the heart of this exploration lies the concept of SE, which is pivotal for understanding how university students' engagement in artistic expression contributes to the cultivation of this crucial aspect of their psychological wellbeing (Ran et al., 2022). Given the nature of EI in young adults, the present study tries to unravel interconnections of EI, artistic expression, and SE within the educational environment of Chinese university system. Emphasizing their cumulative impact on university students' psychological wellbeing, this research aims to provide nuanced insights into the intricate interplay of these psychological constructs. In this regard, the present study frames one hypothesis as follows:

**H4:** Engagement in artistic activities is significantly influenced by emotional intelligence, impacting the self-efficacy of university students.

**Figure 1:**
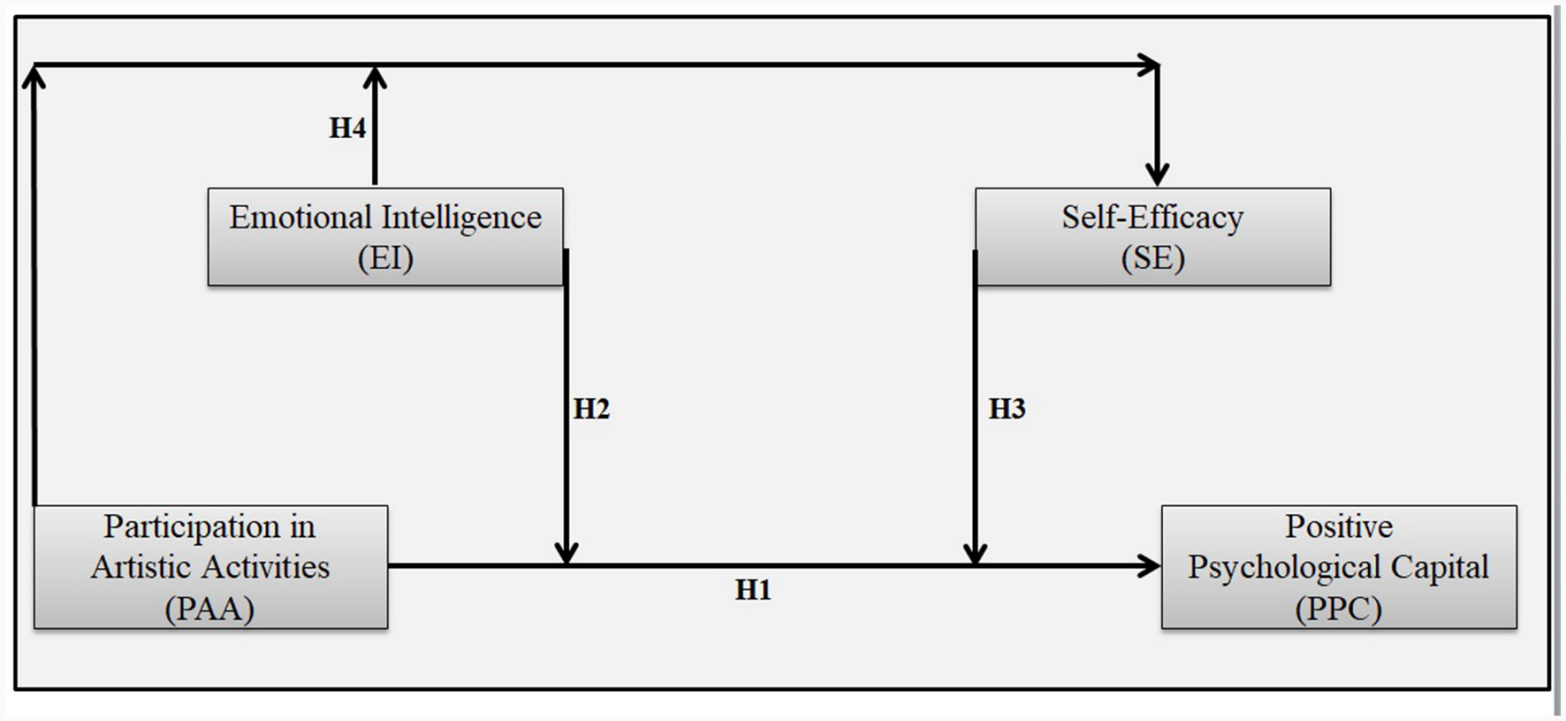
Interconnecting different aspects of current research.

Figure 1 provides a graphical illustration of all these interconnections.

## 4 Methodology

This study employs some well-used software, such as *SPSSStatistics* 29.0 and *bootstrap* for data analysis, while *AMOS* 25.0 software is used for making structural equation modeling (SEM) and confirmatory factor analysis (CFA).

### 4.1 Description of participants

This study focused on the students from nine Chinese universities, which were primarily located in Guangxi and Shanxi provinces. They belonged to various majors, such as medicine, science and technology, arts, humanities and social sciences, and physical education. Distribution of questionnaires took place from July 2023 to September 2023. This was facilitated by the teachers and/or counselors in each place. The collection of online questionnaires ensured confidentiality for the participating students. Out of the 673 questionnaires, this study considered 630 to be valid for analysis after excluding unqualified responses. This resulted in 93.61% questionnaire recovery rate.

### 4.2 Data measurements

Artistic activities are of two types, such as the art viewing and the art participation activities. Art viewing activities encompass actions related to viewing art performances, including visual engagement with exhibitions, museums, and similar venues. Art participation activities have direct involvement in artistic performances, including literary creation/reading, literary activity, engagement in fine arts, and traditional art forms directly associated with creative endeavors and artistic performances. This study amalgamates those two areas and thereby create the university arts activity participation (UAAP)-scale consisting of 16 questions.

Regarding the scales, this study takes into account the scale framed by Luthans and Youssef-Morgan (2017) as positive psychological capital scale (PPCS). This scale is predominantly associated with job and goal attainment within organizational contexts. The scale comprises four dimensions, each featuring 10 question items. Also, this extends beyond university students and the general population to have a domain extensively examined for its reliability.

The EI scale that is to be utilized in this study has been formulated by Wong and Law (2002). This comprises four dimensions, such as emotional regulation, emotional application, self-emotional understanding, and emotional understanding for others. While fellow researchers adapt this since long, this scale demonstrates commendable reliability. Also, this scale incorporates a total of eight questions.

### 4.3 Control variables

The present study exercised control over certain demographic variables, such as grade, gender, and profession of the students. This study adhered to a standard practice observed in numerous existing articles, which involved controlling some standard factors such as gender, age, educational background, and job position. However, this is important to find that current scope lied in a focused approach and was limited to the specific variables considered for control. Specifically, this study took gender to be dummy variable with 1 representing male and 2 representing female. Therefore, this study could acknowledge the potential influence of aforementioned demographic factors.

### 4.4 Descriptive analysis

On basis of the information gathered from 630 university students in China, this study performs a descriptive statistical analysis regarding their demographic characteristics. Among the respondents, this study found 52.38% participants to be male and rest 47.62% to be female. A considerable proportion of these two grades (about 85.71% combined) was such that over 50% students were in *first year* of undergraduate courses, with other 31.57% students in *second year*. Among the different major subjects opted by those students, *science and engineering* accounted for 42.85%, while *arts and sports* was the most popular major having 45.24% of participants. Table 1 enlists the comprehensive statistics in this regard.

**Table 1:** Description of participants.

**Demographic variables**	**Types**	**Frequencies**	**Ratios (%)**
Gender	Male	330	52.38
		Female	300	47.62
Grades	First year	312	49.52
		Second year	180	28.57
		Third year	61	9.68
		Fourth year	67	10.79
		Graduate	10	1.44
Major subject areas	Humanities and social science	270	42.85
	Arts and sports	285	45.24
	Engineering and science	43	6.82
	Medicine	32	5.08

### 4.5 Data validation testing

This study employed the Cronbach's α-test and descriptive statistics. Also, this study analyzed the data with SPSS software for each variable. This study provided the results in Table 2 that showed Chinese university students' participation in diverse artistic activities at a low rate, with a mean of 1.219 and a median of 1.86. Both of these values were lower than 3.

**Table 2:** Status of participants with the reliability test.

	**Mean**	**Standard deviation**	**Median**	**Cronbach α for variables**
PAA	1.219	0.793	1.86	0.872
PPC	3.718	0.710	3.91	0.817
Self-efficacy	3.816	0.713	3.69	0.891
Emotional intelligence	3.618	0.920	3.61	0.819

Again, the mean and median values for various PPC, EI, and SE measures in this study were exceeding 3. This established that the survey participants had high levels of PPC, EI, and SE. Moreover, Cronbach's α values were found to exceed 0.7 for each of the variables. This would advocate for a superior survey reliability.

The present study adopted the validation factor analysis for assessing convergent and discriminant validity for questionnaire. Validation factor analysis (CFA) as measured in AMOS software yielded satisfactory model fit results: $\frac{\chi^{2}}{df}=3.303\left(<5\right)$, CFI = 0.919, NFI = 0.906, and IFI = 0.916 (these values were more than 0.9), SRMR = 0.044(<0.05), and RMSEA = 0.0517(<0.08). Therefore, current results collectively could indicate that the data aligned well with the validation factor analysis model.

On the other hand, this study applied some well-established approaches, such as the standardized factor loading and combination reliability together with the mean variance extraction and arithmetic square roots. Then, this examined the convergent validity of the assembled data. Average variance extracted (AVE) value of any variables exceeded 0.5, while their composite reliability (CR) values were more than 0.7. Table 3 displays the results.

**Table 3:** Convergent plus discriminant validity testing.

	**AVE**	**CR**	**PAA**	**PPC**	**Emotional intelligence**	**Self-efficacy**
PAA	0.616	0.883	0.789			
PPC	0.626	0.869	0.673	0.782		
Emotional intelligence	0.702	0.872	0.618	0.269	0.817	
Self-efficacy	0.635	0.863	−0.052	0.592	0.002	0.794

Aforementioned findings corroborate the questionnaire's qualified convergent validity. Correlation coefficients of variables were then compared to square root of AVE values. Furthermore, this study found that the AVE values along the diagonal were consistently greater than any correlation coefficient in their respective columns. This phenomena demonstrated the discriminant validity of the data, thus establishing the questionnaire validity too.

This study used the one-way confirmatory factor analysis (CFA) and thus assessed the common method bias, while AMOS software was used to perform validation factor analysis on the collected data. Because this study found $\frac{\chi^{2}}{df}=17.993$ (with χ^2^ = 18606.013 being much higher than the study model of 3076.152) to be much more than 5, CFI = 0.521 being considerably lesser than 0.9, IFI = 0.514 being smaller than 0.9 and SRMR = 0.169 being more than 0.1 and RMSEA = 0.166 being more than 0.1, the results of the one-way model were barely considerable. Also, this implied that the current data was free of common method bias.

### 4.6 Testing of the proposed hypotheses

With the aim to indicate whether variables were correlated with each other, this study performs the Pearson correlation analysis on variables in the SPSS software. Table 4 displays the associated results.

**Table 4:** Correlation analysis of the data.

	**Gender**	**Grade**	**Major**	**PAA**	**PPC**	**EI**	**Self-efficacy**
Gender	1						
Grade	0.053	1					
Major	−0.219^#^	−0.347^#^	1				
PAA	0.013	−0.069	0.162^#^	1			
PPC	0.121^#^	−0.079^*^	0.123^#^	0.643^#^	1		
Emotional intelligence	0.120^#^	−0.039	−0.016	−0.053	0.279^#^	1	
Self-efficacy	0.051	−0.068	0.141	0.684^#^	0.512^#^	0.002	1

^#^*p* < 0.01 and ^*^p < 0.05.

This study finds that thee correlation coefficients between SE and PAA (0.684), along with those between SE and PPC (0.512) exceeded zero. Since those were found as significant, the present study could establish a link between SE with PAA and PPC both. Moreover, the correlation coefficient between PPC and PAA was found to be 0.643 with a significance level 0.01. Thus, there exists major positive association between artistic activities and PPC for Chinese university students. Therefore, the results supported the hypothesis **H1**.

On basis of the mediation path PAA → PPC → SE bootstrap test, current research found the direct and total effects both were lesser than 0.001. The total effect value in PAA and SE was accounted for by the indirect effect value for PPC of 0.1009 (LLCI = 0.0255, ULCI = 0.1792, interval exclusion 0). Thus, this study implied that PPC had a partial mediation effect and mediated the relationship between PAA and SE. Therefore, PAA would influence SE both directly and indirectly through PPC, thereby establishing the proposed hypothesis **H3**. Table 5 displays the resulting data.

**Table 5:** Measuring impacts of positive psychological capital of participants.

**Independent variable**	**Dependent variable**	**Influence type**	**Effect**	**BootSE**	**BootLLCI**	**BootULCI**	**Effect ratio**
PAA	SE	Indirect effect	0.109	0.038	0.0255^#^	0.1792	17.12%
		Direct effect	0.479	0.509	0.4339	0.6124	
		Total effect	0.593	0.048	0.503	0.689	

*#p* < 0.001.

Then, this study conducted stratified regression analysis to assess stability of this model. This employed two models in hierarchical regression analysis. Model 1 included independent variables: major, grade, and gender while Model 2 incorporated PAA and PPC in addition to those variables of Model 1. SE was taken as dependent variable in both models. On basis of the results as displayed in Table 6, this study found interaction term product coefficient β for EI and PAA was 0.041 under confidence interval (LLCI = −0.004, ULCI = 0.089) with zero. This showed some moderating effects of EI on relationship between PAA and SE for the Chinese university students to be insignificant. Furthermore, this implied that hypothesis **H4** failed to hold.

**Table 6:** Measuring impacts of emotional intelligence of participants.

**Dependent variables**		**PPC**	**Self-efficacy**			
	**B**	**LLCI**	**ULCI**	β **(EI & PAA)**	**LLCI**	**ULCI**
PAA × EI	0.155^#^	0.119	0.188	−0.034	−0.069	0.01
R2		0.513			0.462	
F		122.07			93.881	

*#p* < 0.001.

However, the data of Model 2 demonstrated how EI moderated relationship between PAA and PPC. This was a clear indication that EI had some significant impacts on magnitude of effects at different levels regarding the effects of PAA participation on PPC. Here, linear relationship between participation in PAA and PPC was flatter at low levels of EI for Chinese university students. Nevertheless, if EI was at high level, slope of the linear relationship between PAA and PPC was determined as longer in comparison with low level. Thus, the resulting moderating effects supported hypothesis **H2**.

## 5 Results and insights

The present study develops a number of actionable insights of basis of current results as follows.

### 5.1 Actionable insights

This study finds the impacts of participation in artistic activities (PAA) over the self-efficacy (SE) for Chinese university students, along with mediating effects of the positive psychological capital (PPC) and moderating effects of the emotional intelligence (EI), thus yielding the following acumen:

- *Positive impacts of arts education over the SE:* Impact of participation in various artistic activities on the SE of Chinese university students is a noteworthy aspect of this study. By actively involving themselves in various artistic pursuits, Chinese university students can experience a range of emotions and psychological fulfillment. Beyond the immediate joys of creative expression, this engagement fosters a sense of fulfillment and satisfaction, contributing to the overall wellbeing of students. Also, this contributes significantly to their overall sense of fulfillment and satisfaction.
- This study determines some significant impacts of Chinese students' active participation in various artistic activities over their lives. The immersive experience to get engaged in various forms of artistic expressions provides an avenue for leisure, while emerging as a potent tool for overcoming challenges. All these consequently cultivate a heightened sense of SE among university students.
- Within the realm of artistic engagement, Chinese university students can find a platform to develop and showcase their unique abilities. This leads to positive impact on their perceptions of personal competence. Moreover, the acknowledgment of achievements in artistic sphere becomes a catalyst for bolstering confidence and reinforcing a positive self-perception for young Chinese adults.
- Apart from several immediate benefits of creativity and leisure associated with artistic pursuits, this study establishes some profound and transformative power inherent in these activities for shaping SE of university students. In essence, this study pleads to portray the various forms of artistic engagements as a dynamic force that shall contribute significantly to personal growth beyond the boundaries of conventional creative expressions.
- *Mediating role of PPC:* Current results show that engaging in artistic activities influences the SE of Chinese university students significantly. This is beyond the immediate joys of creative expression. PPC is an important intermediary in this relationship that includes important elements including self-assurance, optimism, and resilience. This way, PPC plays a major role in mediating the beneficial effects of engaging in artistic pursuits on SE.
- In this regard, the pervasive influence of AI on Chinese university students' lives raises concerns about its associated negative impact. Typically, university students are sometimes exposed to hidden negative information that subsequently lead to a potential deviation in values. Balancing the benefits of AI with the wellbeing of the young adults is essential for maintaining positive psychological states amid evolving technological landscapes.
- Moreover, the participation in various artistic endeavors creates a conducive environment for the development of PPC among students. These contribute to the enhancement of self-confidence by instilling in students a belief in their own abilities. Additionally, the optimistic outlook fostered by the engagement in arts plays a pivotal role in shaping a positive psychological capital. This further influences their sense of SE. This way, the present study establishes the transformative power of artistic activities in shaping the SE of Chinese university students. Also, this puts more emphasis on their participation beyond the realms of creativity and leisure.
- *Moderating role of EI:* Current results show that EI has a crucial moderating role in shaping the intricate dynamics between PAA and PPC among Chinese university students. Those with heightened EI exhibit a superior ability to leverage emotional expression and comprehension within artistic pursuits. This emphasizes the nuanced ways in which EI augments the emotional dimensions inherent in these activities.
- Contrary to initial expectations, the study findings dismiss the hypothesis proposing that EI directly moderates the relationship between PAA and SE for Chinese university students. Instead, the results suggest that impact of EI on the choices related to artistic activities is, at most, indirect. Despite this, this study underscores the profound influence of EI on students' engagement with the emotional facets embedded in these activities. Thus, while not directly shaping SE, EI significantly shapes the emotional landscape in which students participate in artistic pursuits. This nuanced interplay highlights the multifaceted nature of EI in the context of Chinese university students' involvement in diverse creative expressions.
- *Other implications:* This study contributes to the theoretical insights influencing Chinese university students' SE, while unraveling the interconnections among PAA, PPC, and EI. Grounded in a real-life perspective, these results advocate for some strategic interventions by Chinese universities to bolster students' SE through a multifaceted approach encompassing artistic activities, EI, and PPC development, contributing to a more comprehensive and student-centric educational experience.
- This study advocates for Chinese universities to play a pivotal role in enhancing students' SE by actively promoting engagement in artistic pursuits. By providing resources for participation in these activities, Chinese universities can contribute to the students' emotional experiences while fostering a sense of fulfillment and meaning. Recognizing the mediating role of PPC and moderating influence of EI, they can also design some effective programs to nurture the psychological resources.
- The proposed approach should fortify emotional wellbeing and resilience for Chinese university students, thus positively impacting their SE. Researchers describe these as vital for the development of SE among young adults. The emphasis on the importance of artistic activities for the mental health and overall wellbeing of Chinese university students seek some specific support strategies. Administrations across colleges and universities should consider the integration of artistic engagements into their broader wellbeing initiatives.

#### 5.1.1 Global aspects

Taking the aforementioned China specific insights into consideration, this study further pleads to frame the discussion on arts education within the context of international perspectives. The immersive experience provided by artistic activities, fostering a profound sense of fulfillment and satisfaction, transcends cultural boundaries. Therefore, at the international level, current results underscore the global significance of arts education to enhance the wellbeing and self-efficacy of young adults. Advocating for the promotion of artistic engagement as one universal policy, this study calls for a collective acknowledgment of the transformative potential that arts education holds for fostering emotional wellbeing and SE among university students.

The study emphasizes the need for educational institutions worldwide to integrate artistic engagements into broader wellbeing initiatives. By adopting this approach, those institutions of higher learning can contribute to a more comprehensive and enriched educational experience for students across diverse cultural contexts. Moreover, this approach recognizes the universal importance of arts education as a dynamic force for personal growth and wellbeing on an international scale.

### 5.2 Limitations of the present study

Current research, like any systematic investigation in this domain, has several limitations. Firstly, this study uses a cross-sectional design restricting the establishment of causal relationship. A reliance over the self-reported data brings chances of biased response. Students sometimes offer socially favorable answers or remember their engagement in artistic activities, emotional experiences, or psychological states inaccurately. The subjective nature of self-reporting should be considered when interpreting those acumen.

This study gathered data mainly from universities in Shanxi and Guangxi provinces, thus being a predominantly regional sample. This regional focus can hardly represent the diversity of Chinese university students. Thus, this necessitates caution when extrapolating conclusions to a broader population with potential regional and cultural variations. Again, while this study controlled for demographic factors such as gender, grade, and major, the influence of other relevant variables remains unexplored. Moreover, the amalgamation of art viewing and art participation activities into a single scale may oversimplify the measurement of artistic activities. Different art forms could have distinct effects on psychological outcomes, and a more detailed analysis could provide valuable insights into the specific mechanisms at play. Considering the unique challenges posed by the COVID-19 pandemic, this study has failed to extensively address its influence is a notable limitation.

## 6 Conclusions

The present study successfully uncovers the intricate mechanisms influencing the SE of Chinese university students through an exploration of the interplay among PAA, PPC, and EI. The results affirm the positive impacts of engaging in artistic activities on students' SE, highlighting the transformative power of artistic pursuits beyond mere leisure. PAA has played a vital role in shaping cognitive, emotional, and behavioral patterns, contributing to a sense of fulfillment and satisfaction. Moreover, this study shows the mediating role of PPC, emphasizing how the cultivation of self-confidence and resilience through artistic engagement channels the positive influence on SE, underscoring the holistic impact on psychological wellbeing. Additionally, this study determines the moderating role of EI in the dynamics between PAA and PPC, highlighting its nuanced influence on the emotional landscape during artistic engagement.

The theoretical implications extends to a broader understanding of the relationships between arts education, EI, and SE. This study advocates for a holistic approach in educational institutions, emphasizing the integration of artistic engagement into wellbeing initiatives. This underscores the transformative potential of arts education, particularly in the face of AI-related technological advancements and the evolving landscape of higher education. While indirectly influencing the relationship between artistic engagement and SE, EI significantly shapes the emotional landscape in which students engage in artistic pursuits. This nuanced interplay emphasizes the multifaceted nature of EI in the context of Chinese university students' involvement in diverse artistic expressions.

In light of these findings, this study emphasizes the pivotal role of universities in fostering students' SE by actively promoting engagement in artistic pursuits. Recognizing the mediating role of PPC and the moderating influence of EI, universities should design targeted programs to nurture these psychological resources, thereby enhancing students' emotional wellbeing and resilience. Current emphasis on the importance of artistic activities for mental health advocates for specific management and support strategies within educational institutions. As Chinese higher education undergoes transformations, this turns essential to investigate the profound impact of arts education to shape an enriching educational experience for university students.

### 6.1 Scopes of future research

Future researchers can extend this study in several different directions. Researchers can consider a longitudinal approach to explore the long-term impact of cultural engagement on emotional wellbeing and self-perceived capabilities. Exploring regional cultural variations within China and delving into the specific influence of different art forms on the PPC, EI, and SE can offer promising avenues. Also, this is essential to explore the impact of AI on all these. One can design some interventions or educational programs that leverage artistic activities. This will enhance the wellbeing among university students. Additional exploration on how individuals with higher SE are more inclined to participate in artistic pursuits can unveil additional insights into the reciprocal nature of these relationships, informing the development of targeted interventions for specific student groups. Diversifying the research scopes beyond China to a global or other geographical contexts offers a promising area for further exploration. This expansion shall enhance the generalizability and applicability of insights, contributing to a more comprehensive understanding of the subject matter.

## Data availability statement

The raw data supporting the conclusions of this article will be made available by the authors, without undue reservation.

## Ethics statement

Written informed consent was obtained from the individual(s) for the publication of any potentially identifiable images or data included in this article.

## Author contributions

YG: Writing—original draft, Writing—review & editing.
